# A comprehensive examination of the lysine acetylation targets in paper mulberry based on proteomics analyses

**DOI:** 10.1371/journal.pone.0240947

**Published:** 2021-03-11

**Authors:** Ping Li, Chao Chen, Ping Li, Yibo Dong

**Affiliations:** 1 College of Animal Science, Guizhou university, Guiyang, Guizhou, China; 2 Institute of Grassland Research, Sichuan Academy of Grassland Science, Cheng Du, Si Chuan, China; Universita degli Studi di Milano, ITALY

## Abstract

Rocky desertification is a bottleneck that reduces ecological and environmental security in karst areas. Paper mulberry, a unique deciduous tree, shows good performance in rocky desertification areas. Its resistance mechanisms are therefore of high interest. In this study, a lysine acetylation proteomics analysis of paper mulberry seedling leaves was conducted in combination with the purification of acetylated protein by high-precision nano LC-MS/MS. We identified a total of 7130 acetylation sites in 3179 proteins. Analysis of the modified sites showed a predominance of nine motifs. Six positively charged residues: lysine (K), arginine (R), and histidine (H), serine (S), threonine (T), and tyrosine (Y) occurred most frequently at the +1 position, phenylalanine (F) was both detected both upstream and downstream of the acetylated lysines; and the sequence logos showed a strong preference for lysine and arginine around acetylated lysines. Functional annotation revealed that the identified enzymes were mainly involved in translation, transcription, ribosomal structure and biological processes, showing that lysine acetylation can regulate various aspects of primary carbon and nitrogen metabolism and secondary metabolism. Acetylated proteins were enriched in the chloroplast, cytoplasm, and nucleus, and many stress response-related proteins were also discovered to be acetylated, including PAL, HSP70, and ERF. HSP70, an important protein involved in plant abiotic and disease stress responses, was identified in paper mulberry, although it is rarely found in woody plants. This may be further examined in research in other plants and could explain the good adaptation of paper mulberry to the karst environment. However, these hypotheses require further verification. Our data can provide a new starting point for the further analysis of the acetylation function in paper mulberry and other plants.

## Introduction

Ecosystem degradation and soil erosion are major problems that restrict the regional economy in karst areas. Ecological restoration is a focus in these regions, and the selection of suitable vegetation can be challenging because of these problems. The strong ability of paper mulberry to withstand environmental pressures indicates that further exploration of its resistance mechanisms is necessary.

Proteins, as important type components encoded by genes that control the entire metabolic process. Protein regulation encompasses multilayered and interconnected transcriptional and translational processes [1]. This process begins with transcription from DNA and the splicing of genes into RNA molecules, which are subsequently translated into polypeptides later [2]. However, in the absence of posttranslational modifications, proteins likely cannot perform as many functions. Posttranslational modifications (PTMs) of histone are well-known for their critical roles in cellular pathways, as they can change the physicochemical properties of proteins and affect their activity and stability [3, 4]. When PTM occurs to a protein, it will directly change the protein’s binding ability and function, introducing new functions by introducing new functional groups such as acetyl, phospho, ubiquityl, succinyl and methyl groups [5]. Among PTMs, lysine acetylation was first discovered on histone tails where chromatin structure and gene expression are regulated. Changes in cellular lysine acetylation status can also alter metabolic enzyme activity and provide an adaptive mechanism for specific metabolic changes in cells [6].

Paper mulberry (*Broussonetia papyrifera*) is a unique fiber-bearing economic forest species in China due to its wide distribution, fast growth, strong adaptability and resistance to extreme environments. In recent years, it has been continuously developed and applied in different fields, especially as leaves for animal feed and bark and branches as sources of advanced papermaking materials and medicines. Based on transcriptomics, proteomics, phosphorylated proteomics and glycoproteomics studies, whole-genome analysis is now widely applied among archaea, bacteria and real bacteria. The acetylation of proteins in all three life fields, including nuclear biology, has been explored [7, 8]. Some studies have been performed on lysine acetylation, and many histone acetyl transferases [9–11] have been identified in plants. Many studies have also shown that histone acetylation is involved in the responses to different environmental factors, including light and low temperature, to help plants better adapt to extreme conditions. It is evident that protein acetylation plays a much broader role than merely regulating of histone functions [12]. At the same time, qualitative, quantitative and functional analysis of key proteins in specific key metabolic pathways can be performed [13]. An interaction between the protein acetylation modification in paper mulberry and its anti-stress mechanism has been reported, but the types of acetylases in paper mulberry and the acetylation of important biologically-related pathways such as photosynthesis and metabolism remain to be further explored. Moreover, the modifications and expression patterns are unclear.

In this study, we aimed to identify systematically characterize lysine acetylation sites in tree proteins. This research will enhance our understanding of how paper mulberry has adapted to extreme karst environments.

## Methods

### Plant materials

The experimental materials were selected from Guizhou in August 2019 at a coordinate longitude of 106.66136 and latitude of 26.45024., and cutting seedlings with the same growth were cultivated by using the cutting propagation technique. The plant grows in greenhouse conditions (22 °C -25 °C) and under natural light cycles. About a month later, when the plant height was approximately 20 cm, 15g of uniformly growing leaves were collected from the vegetative stage of the plant, frozen immediately in liquid nitrogen and stored at -80°C until used for RNA extraction. All experiments were performed at least 3 times using independently collected and extracted tissues, unless otherwise indicated.

#### Protein extraction

Each sample was ground in liquid nitrogen into cell powder and then transferred to a 5-mL centrifuge tube. After that, four volumes of lysis buffer (8 M urea, 1% Triton-100, 10 mM dithiothreitol, and 1% Protease Inhibitor Cocktail) were added to the cell powder, followed by sonication three times on ice using a high-intensity ultrasonic processor. The remaining debris was removed by centrifugation at 20,000 × g at 4 °C for 10 min. Finally, the protein was precipitated with cold 20% TCA for 2 h at -20 °C. After centrifugation at 12,000 × g at 4 °C for 10 min, the supernatant was discarded. The remaining precipitate was washed with cold acetone three times. The protein was redissolved in 8 M urea and the protein concentration was determined with a BCA Kit according to the manufacturer’s instructions.

#### Trypsin digestion

For digestion, the protein solution was reduced with 5 mM dithiothreitol for 30 min at 56 °C and alkylated with 11 mM iodoacetamide for 15 min at 25 °C in darkness. The protein sample was then diluted by adding 100 mM TEAB to a urea concentration less than 2 M. Finally, trypsin was added at a 1:50 trypsin-to-protein mass ratio for the first digestion overnight and at a 1:100 trypsin-to-protein mass ratio for a second 4 h digestion.

#### TMT/iTRAQ labeling

After trypsin digestion, the peptide was desalted with a Strata X C18 SPE column and vacuum-dried. The peptide was then reconstituted in 0.5 M TEAB and processed with the TMT Kit/iTAQ Kit according to the manufacturer’s protocol for the TMT Kit/iTRAQ Kit.

#### HPLC fractionation

The tryptic peptides were fractionated by high pH reverse-phase HPLC using a Thermo Betasil C18 column (5 μm particles, 10 mm ID, 250 mm length). Briefly, the peptides were first separated with a gradient of 8% to 32% acetonitrile (pH 9.0) over 60 min into 60 fractions. Then, the peptides were combined and dried by vacuum centrifugation.

#### LC-MS/MS analysis

The tryptic peptides were dissolved in 0.1% formic acid and directly loaded onto a homemade reversed-phase analytical column (15-cm length, 75 μm i.d.). The gradient consisted of an increase from 6% to 23% solvent B (0.1% formic acid in 98% acetonitrile) for 26 min, 23% to 35% for 8 min, climbing to 80% over 3 min and the holding at 80% for the last 3 min, all at a constant flow rate of 400 nL/min using an EASY-nLC 1000 UPLC system.

The peptides were subjected to an NSI source followed by tandem mass spectrometry (MS/MS) in a Q ExactiveTM Plus (Thermo) coupled online to the UPLC. The applied electrospray voltage applied was 2.0 kV. The m/z scan range was 350 to 1800 for a full scan, and intact peptides were detected in the Orbitrap at a resolution of 70,000. Peptides were then selected for MS/MS using the NCE setting of 28, and the fragments were detected in the Orbitrap at a resolution of 17,500. The data-dependent procedure that alternated between one MS scan followed by 20 MS/MS scans with 15.0 s dynamic exclusion. Automatic gain control (AGC) was set at 5E4. The fixed first mass was set to 100 m/z.

#### Database search

The resulting MS/MS data were processed using the Maxquant search engine (v.1.5.2.8). Tandem mass spectra were searched against a database concatenated with a reverse decoy database. The protein sequence database is derived from the transcriptome sequencing data of previous studies (sequences: 25,412). Trypsin/P was specified as a cleavage enzyme, and up to 4 missing cleavages were allowed. The mass tolerance for precursor ions was set as 20 ppm in the first search and 5 ppm in the main search, and the mass tolerance for fragment ions was set to 0.02 Da. Carbamidomethyl on Cys was specified as a fixed modification, acetylation and oxidation on Met were specified as variable modifications. The FDR was adjusted to < 1%, and the minimum score for modified peptides was set to > 40.

#### Domain annotation

Identified protein domain functional descriptions were annotated by InterProScan based on the protein sequence alignment method, and the InterPro domain database was used (http://www.ebi.ac.uk/interpro/).

### Functional enrichment

#### Gene ontology enrichment analysis

Proteins were classified by GO annotation into the following three categories: biological process, cellular component and molecular function. For each category, a two-tailed Fisher’s exact test was employed to test the enrichment of the identified modified protein against all proteins from the species database. The GO terms with a corrected p-value < 0.05 were considered significant.

#### Pathway enrichment analysis

The Kyoto Encyclopedia of Genes and Genomes (KEGG) database was used to identify enriched pathways using a two-tailed Fisher’s exact test to test the enrichment of the identified modified protein against all proteins in the species database. A pathway with a corrected p-value < 0.05 was considered significant. These pathways were classified into hierarchical categories according to the KEGG website.

#### Protein domain enrichment analysis

For each category of proteins, the InterPro (a resource that provides functional analysis of protein sequences by classifying them into families and predicting the presence of domains and important sites) database was researched, and a two-tailed Fisher’s exact test was employed to test the enrichment of the identified modified protein against all proteins in the species database. Protein domains with a corrected p-value < 0.05 were considered significant.

#### Enrichment-based clustering

For further hierarchical clustering based on differentially modified protein functional classification, we first collated all the categories obtained after enrichment along with their P-value, and then filtered for those categories that were enriched in at least one of the clusters with a P-value <0.05. This filtered P-value matrix was transformed by the function x = −log10 (P-value). Finally, these x values were z-transformed for each functional category. The scores were then clustered by one-way hierarchical clustering in Genesis. Cluster membership was visualized by a heat map using the “heatmap.2” function from the “gplots” R-package.

#### Protein-protein interaction network

All differentially expressed modified protein database accessions or sequences were searched against the STRING database version 10.5 for protein-protein interactions. Only interactions between the proteins belonging to the searched data set were selected, thereby excluding external candidates. STRING defines a metric called the “confidence score” to define interaction confidence; we fetched all interactions that had a confidence score >0.7. The Interaction network from STRING was visualized in the R package “networkD3”.

## Results and discussion

**Fig 1:**
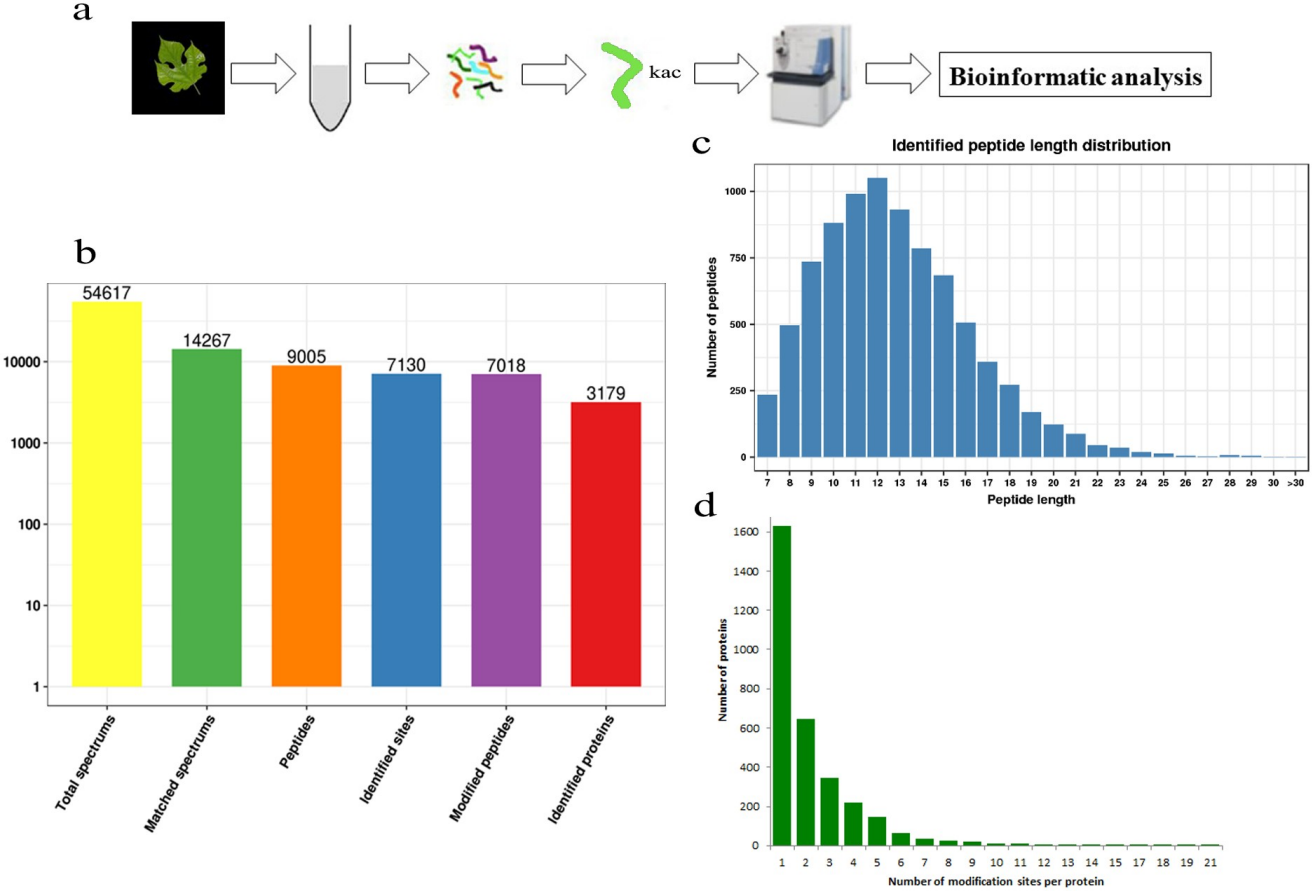
Detection of lysine acetylated proteins in *Broussonetia Papyrifera*. (a) The workflow of integrated strategy for global mapping of lysine acetylation in *Broussonetia papyrifera*. (b) Basic statistical figure of MS results. (c) Length distribution of the peptides. (d) Distribution of the number of the lysine acetylation sites per protein.

Paper mulberry is a perennial tree species characterized by a higher growth rate and greater adaptability to adverse environments than other species. Although paper mulberry is a pioneer tree species in the karst region, we know very little about the resistance mechanisms of this plant at the genomic level. Protein acetylation, a type of PTMs, has been revealed to play critical roles in various physiological processes related to adaptive reactions [14–16]. Therefore, we used PTM technology and LC-MS/MS to systematically express the lysine-acetylated proteins in paper mulberry to further study its adaptive mechanism, and the workflow of experimental procedures used in the study was shown in Fig 1a.

A total of 54617 secondary spectra were obtained by mass spectrometry during the identification (Fig 1b). When the mass spectrometry secondary spectra were searched against the protein theory data, the available efficiency was 14267, the spectrum utilization rate was 26.1%, and the peptides were resolved into 9005 peptides and 7018 acetylated peptides. A total of 7,130 acetylation sites were identified on 3179 proteins in this study and are included in the subsequent analyses (S1 Table).

### Subsequent analysis of the acetylation sites by bioinformatics analysis confirms the modification sites and modified proteins

In this identification, the lengths of the peptides ranged from 7 to 30 amino acids, and most were between 7 and 25 amino acids (Fig 1c). The number of modification sites per protein ranged from 1 to 21 (Fig 1d), and more than 50% of proteins contained only one acetylation site. It is well-known that the histone core is a site for histone acetylation [17], for instance, rice GF14e encodes a 14-3-3 protein that negatively affects cell death and disease resistance in rice [18], and plant 14-3-3 proteins modulate important cellular processes by interacting with a diverse range of target proteins [19]. Additionally, different species may have different protein acetylation levels, sometimes varying significantly. In a comprehensive analysis of protein and nitrogen nutrition, a total of 1286 proteins with lysine in tea plant species were shown to be acetylated [20]; moreover, tea leaves in different periods will have different amounts of protein acetylated [21]. However, as the first lysine acetylation group map of woody plants, this analysis is expected to provide valuable resources for future PTM research.

### Motif characteristics of the acetylated peptides

**Fig 2:**
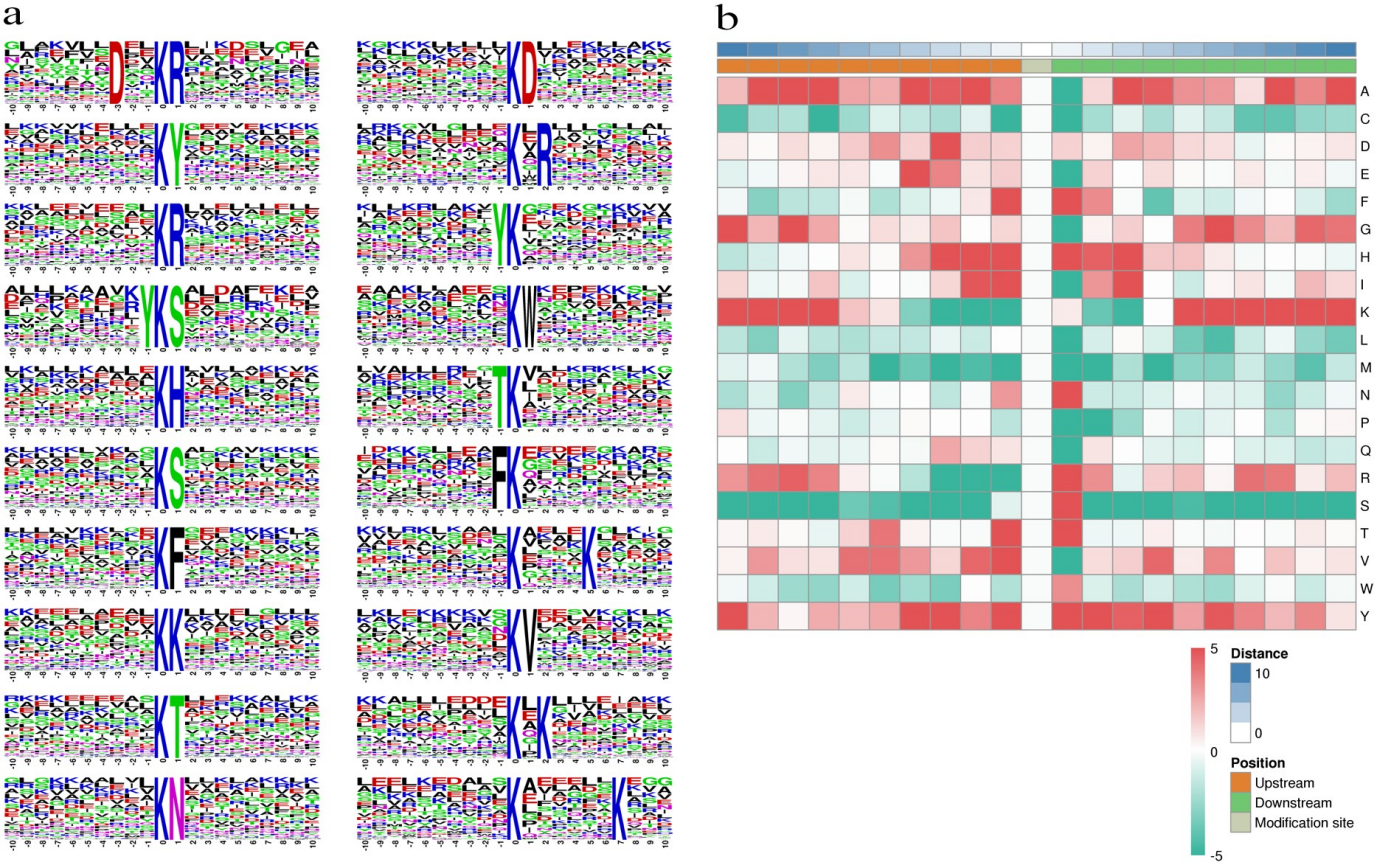
Motif analysis of lysine-acetylated peptides. (a) Sequence probability logs of significantly enriched acetylation site motifs for +10 amino acids around the lysine acetylation sites. (b) The motif enrichment heatmap of upstream and downstream amino acids of all identified modification sites.

To better understand the features of the acetylated sites, the flanking amino acid residues from positions -10 to +10 around the acetylated lysine were analyzed (S2 Table**)**. Motif analysis of the modified sites showed a predominance of 12 motifs, as shown in Fig 2a, including D*K^ac^R, K^ac^*Y, K^ac^*R, Y*K^ac^S, K^ac^*H, K^ac^*S, K^ac^*F, K^ac^*K, K^ac^*T, K^ac^*N, K^ac^*D, Y*K^ac^, K^ac^*W, T*K^ac^, F*K^ac^, and K^ac^*V. Among these motifs (Fig 2a and 2b), the following six positively charged residues: lysine (K), arginine (R), and histidine (H), serine (S), threonine (T), and tyrosine (Y), occurred most frequently at the +1 position, which were all in positively charged. Amino acid biases may reflect a bona fide preference or may be due to the preference of antibodies used for selective enrichment of acetylated peptides [22, 23]. In previous studies, three motifs common in rice (K, H, and F) also existed in the Gram-negative marine bacterium *V*. *parahemolyticus* [24]. phenylalanine (F) was detected both upstream and downstream of the acetylated lysines. F is an essential amino acid and a precursor of thousands of secondary metabolites in animals that cannot synthesize F, indicating that these animals must obtain F directly or indirectly from plants [25]. Moreover, R, H, Y, and S are the most common amino acids in *roseosporus* motifs and *Camellia sinensis* [26, 27], Choudhary et al. also found that amino acids with a bulky side chain (mainly Y and F) were enriched in the -2 and +1 positions in human cells [28]. Therefore, the motif analysis suggests that acetylation preferentially occurs at alkaline and positively amino acids in nearby regions in paper mulberry, may be conserved and are important for lysine acetylation in plants, which may help elucidate the acetylation structure of paper mulberry.

### Functional annotation of acetylated proteins

GO analysis is an important bioinformatics analysis method and tool for determining the expressing various properties of genes and gene products and can explain the biological effects of proteins from different perspectives in paper mulberry. We performed a statistical analysis of the distribution of proteins corresponding to the identified modification sites in the GO secondary annotation.

**Fig 3:**
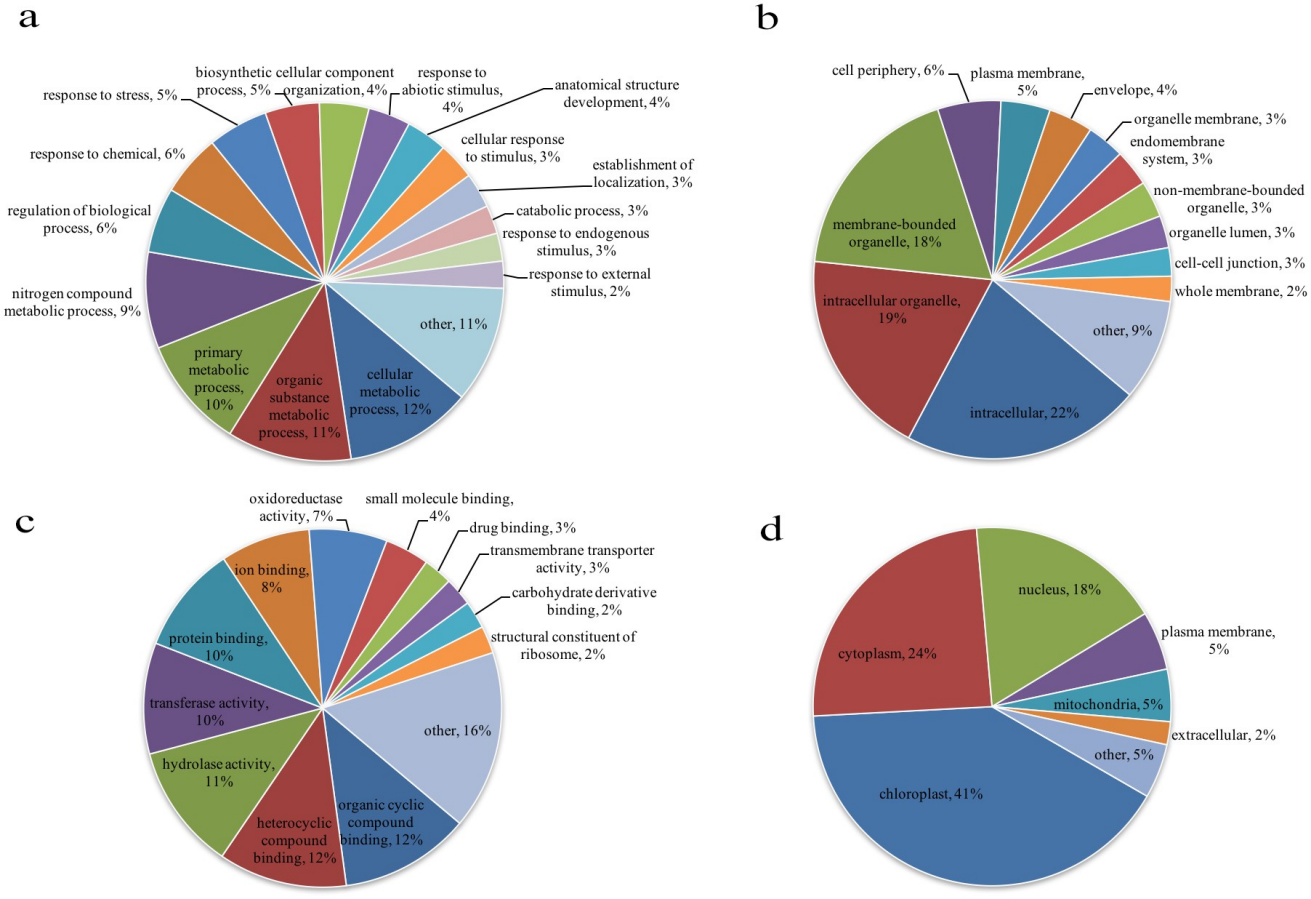
Functional classification of acetylated proteins in *Broussonetia papyrifera*. (a) Classification based on biological process. (b) Classification based on cellular component. (c) Classification based on molecular function. (d) Subcellular location prediction.

In the biological process category (Fig 3a), the most prevalent process GO terms were cellular metabolic process (12%), organic substance metabolic process (11%), and primary metabolic process (10%). It is also worth noting that response to stress accounted for 5%, mainly including salt stress protein and response to oxidative stress protein (S3 Table). Salt stress has an inhibitory effect on plants photosynthesis, and as the external salt concentration increases, the degree of inhibition is greater [29]. This allows us to understand the reasons why paper mulberry can grow well under salinization in karst areas. In addition, the ability of chloroplasts of drought-affected plants to use CO_2_ in carbon assimilation is limited under the light, and therefore energy consumption is reduced [30], the proportion of electrons transferred to O_2_ is relatively increased [31]; thus, O_2_^-^ and H_2_O_2_ can be formed. With metal ion catalysis, it can form more active and aggressive -OH [32–34]. In the face of oxidative stress caused by drought, paper mulberry can provide rich antioxidants for stress proteins, intuitively providing evidence for the resistance of the tree in the molecular function classification. Among the cellular component category, intracellular (22%), intracellular organelle (19%), and membrane-bound organelle (18%) were the main components (Fig 3b). We also observed that most acetylated proteins were related to heterocyclic compound binding (12%), organic cyclic compound binding (12%), hydrolase activity (11%), transferase activity (10%) and protein binding (10%) in the molecular function; others proteins accounted for a large proportion and the remainder accounted for less than 10% (Fig 3c).

According to the functional annotation (S3 Table), enzymes related translation, transcription, ribosomal structure and biogenesis were identified. However, the majority of the enzymes are involved in metabolism-related categories, such as carbohydrate transport and metabolism, amino acid transport and metabolism, and nucleotide transport and metabolism. Early research found that metabolism-related enzymes can play an important role in the regulation of cellular metabolism. The observation of lysine-acetylated substrate protein in bacterial metabolic enzymes verifies the key functional role of such modifications in metabolism [35]. The various mechanisms play diverse role in the regulation of metabolic enzymes [36, 37], and some proteins showing homology between different species have been characterized as being involved in lysine acetylation, indicating their evolutionary and functional conservation [38, 39]. Subcellular localization analysis of acetylated proteins from paper mulberry shows that most proteins are expected to localize to the chloroplast (40.83%), cytoplasm (24.44) % and nucleus (17.71%), while others accounted for only 17.03% (Fig 3d).

**Fig 4:**
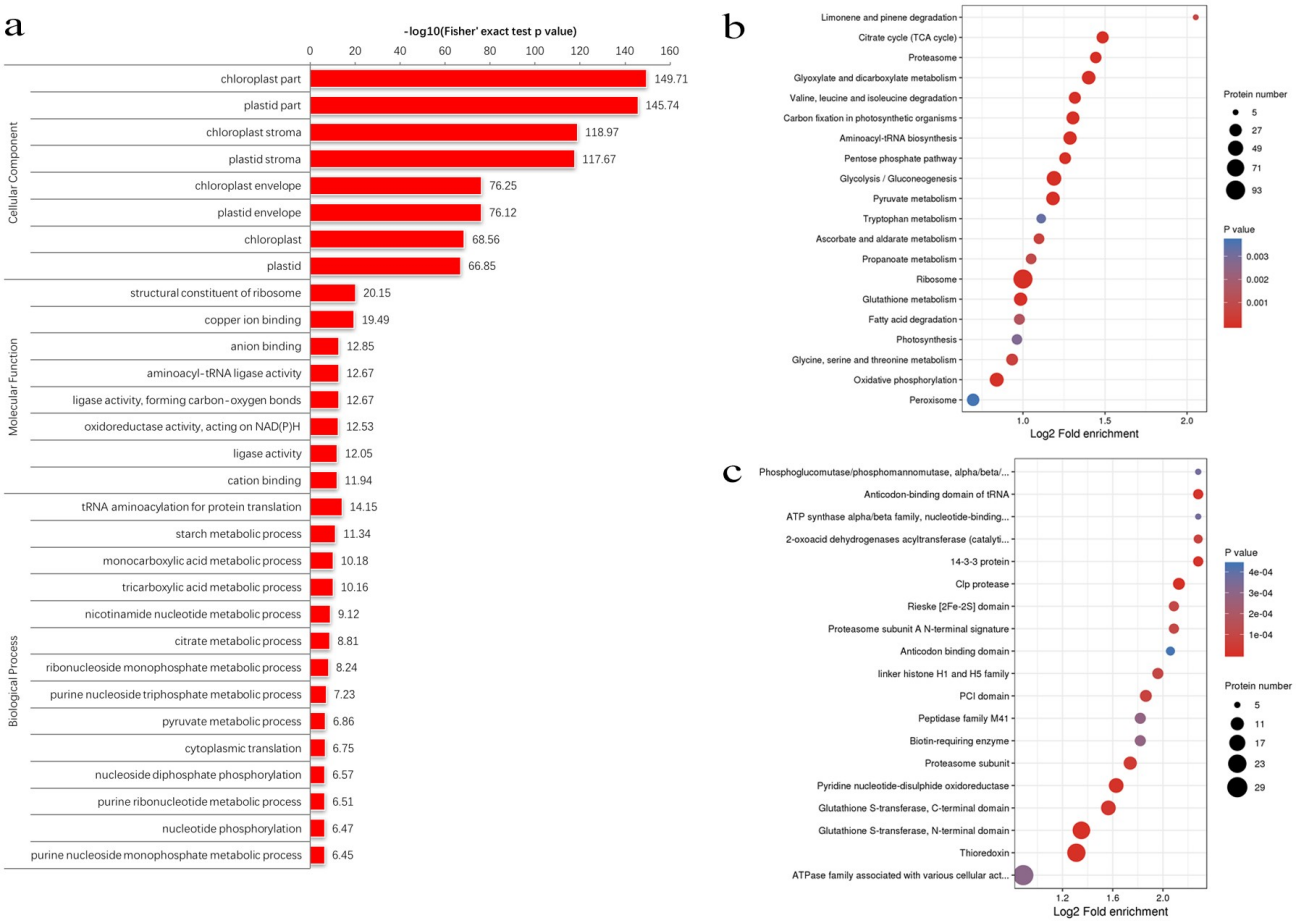
Enrichment analysis of the lysine acetylated proteins in *Broussonetia papyrifera*. (a) Enrichment based on GO annotation. (b) Enrichment based on KEGG pathways. (c) Enrichment based on protein domains.

A protein domain is a conserved part of a given protein sequence and structure that can evolve, function and exist independently of the rest of the protein chain. To better determine the proteins more prone to acetylation, we performed protein domain enrichment analysis on the acetylated proteome to confirm the previous conclusion (Fig 4a). We noticed that some protein complexes, including parts of the chloroplast (≧68.56%) and plastid (≧66.85%), were preferentially acetylated. We learned from other studies that photosynthetic activities can be represented as the growth potentials of the plant [40–43], and that chlorophyll plays an important role in the response of leaf photosynthesis to environmental stresses [44]. Others have suggested that lysine acetylation may be an important posttranslational modification in the chloroplast [45]. Increasing photosynthetic energy-use efficiency and enhancing photosynthetic capacity may be the most successful mechanisms for alien species invasion and adaptability to adverse environments [46, 47]. Meanwhile, structural constituents of ribosomes (20.15%), copper ion binding (19.49%), anion binding (12.85%), aminoacyl-tRNA ligase activity (12.67%), ligase activity, forming carbon-oxygen bonds (12.67%), oxidoreductase activity, and acting on NAD(P)H (12.53%) were also enriched (Fig 4a), which are important in various cellular functions in plants. A total of 20 pathways were enriched in the KEGG enrichment (Fig 4b), including limonene and pinene degradation, TCA cycle, proteasome, glyoxylate and dicarboxylate metabolism, ribosome and so on. The cellular localization and function of proteins are often dictated by their domains [40], among in the identified protein domains (Fig 4c), glutathione-, thioredoxin-, and ATPase-related proteins were significantly enriched.

This result indicates that lysine acetylation tends to target large macromolecular complexes, as has been reported in humans [40], associated with various processes, such as substance transport and metabolism, oxidation-reduction, protein synthesis, and chromatin remodeling [48]. Lysine acetylation also provides material transport and plays roles in metabolism and redox reactions, which can play a role in the regulation of cellular metabolism and stress responses in fraternization. Expansion, antioxidant, metabolic, and detoxification effects may be related to the adaptability of paper mulberry to extreme environments and medicinal functions.

In general, for paper mulberry, lysine acetylation may regulate various aspects of primary carbon and nitrogen metabolism as well as secondary metabolism. However, further confirmation is required. Key acetylated proteins should be purified and selected, and functional studies should be performed to examine what happens following the vitro site-directed mutagenesis of lysine to arginine or glutamine [49–51].

### Abundant lysine acetylation in photosynthesis and phenylalanine metabolism of paper mulberry

Previously large-scale proteomics studies have shown that lysine acetylation is widespread in the mitochondria [52, 53], but the analysis of acetylated proteins from paper mulberry suggesting that the acetylated proteins located in the chloroplast might play important roles in regulating photosynthesis.

**Fig 5:**
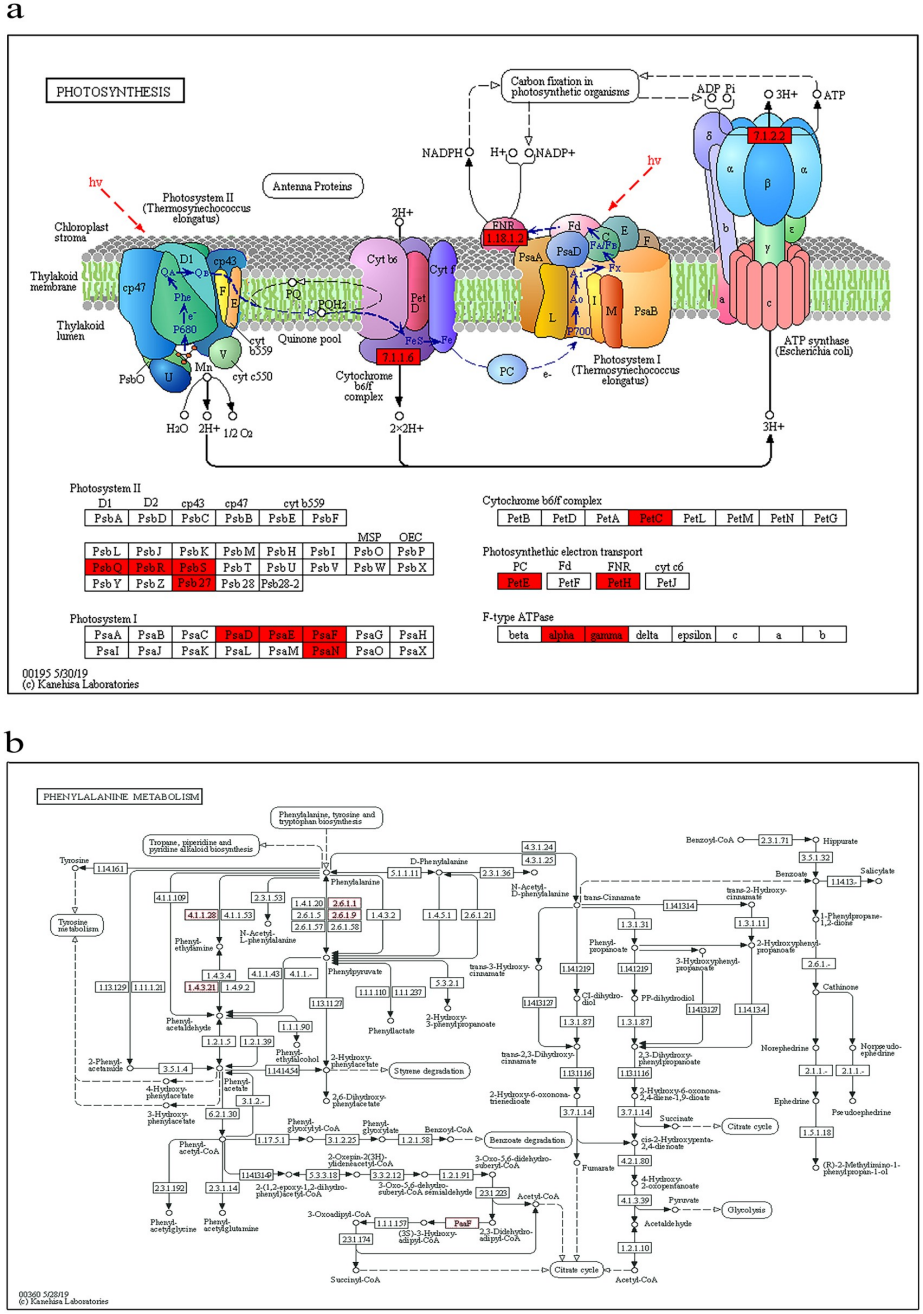
Significantly enriched KEGG pathways. (a) Photosynthesis. (b) phenylalanine metabolism. The acetylated proteins are marked with red. The pictures are drawn by KEGG Mapper. (www.kegg.jp/kegg/tool/map_pathway2.html).

Our study showed that 16 acetylated proteins were involved in photosynthesis (Fig 5a). This process is catalyzed by these multisubunit membrane-protein complexes, including photosystems I and II, the photosynthetic electron transport, and F-type ATPases. Additionally, phenylalanine metabolism plays an important role in plant growth, development and pathological/stress response processes. In the KEGG pathway, there were 6 acetylated proteins in the phenylalanine pathway (Fig 5b), the differentially expressed genes were annotated as phenylalanine ammonia-lyase (PLA), aspartate aminotransferase, histidinol-phosphate aminotransferase, aromatic-L-amino-acid/L-tryptophan decarboxylase, enoyl-coA hydratase and aspartate aminotransferase, mitochondrial, which are key synthase genes in the lignin monomer synthesis pathway. The downstream branch pathways are mainly divided into the flavonoid synthesis pathways and lignin synthesis pathways [54]. Entry into a specificity downstream branch pathway generates a specific metabolic product of benzene propane. For example, the downstream branch pathway promoted the reaction of phenyl propane metabolites, including coumarin, flavonol, lignin, cork vinegar and other benzene compounds (phytoalexin, protection factor, flower and fruit pigment); these structural cell wall components and signaling molecules, such as play a different role in plant growth and development.

To our knowledge, photosynthesis plays a very important role in plant responses to drought stress, and the phenylalanine metabolic pathway is of great significance to mulberry structure, as it also produces a variety of secondary metabolites such as flavonoids, lignin, plant protections, chlorogenic acid, etc. These secondary metabolites have important roles in plant growth and development, disease resistance and adverse reaction resistance.

### Interaction network of acetylated protein

**Fig 6:**
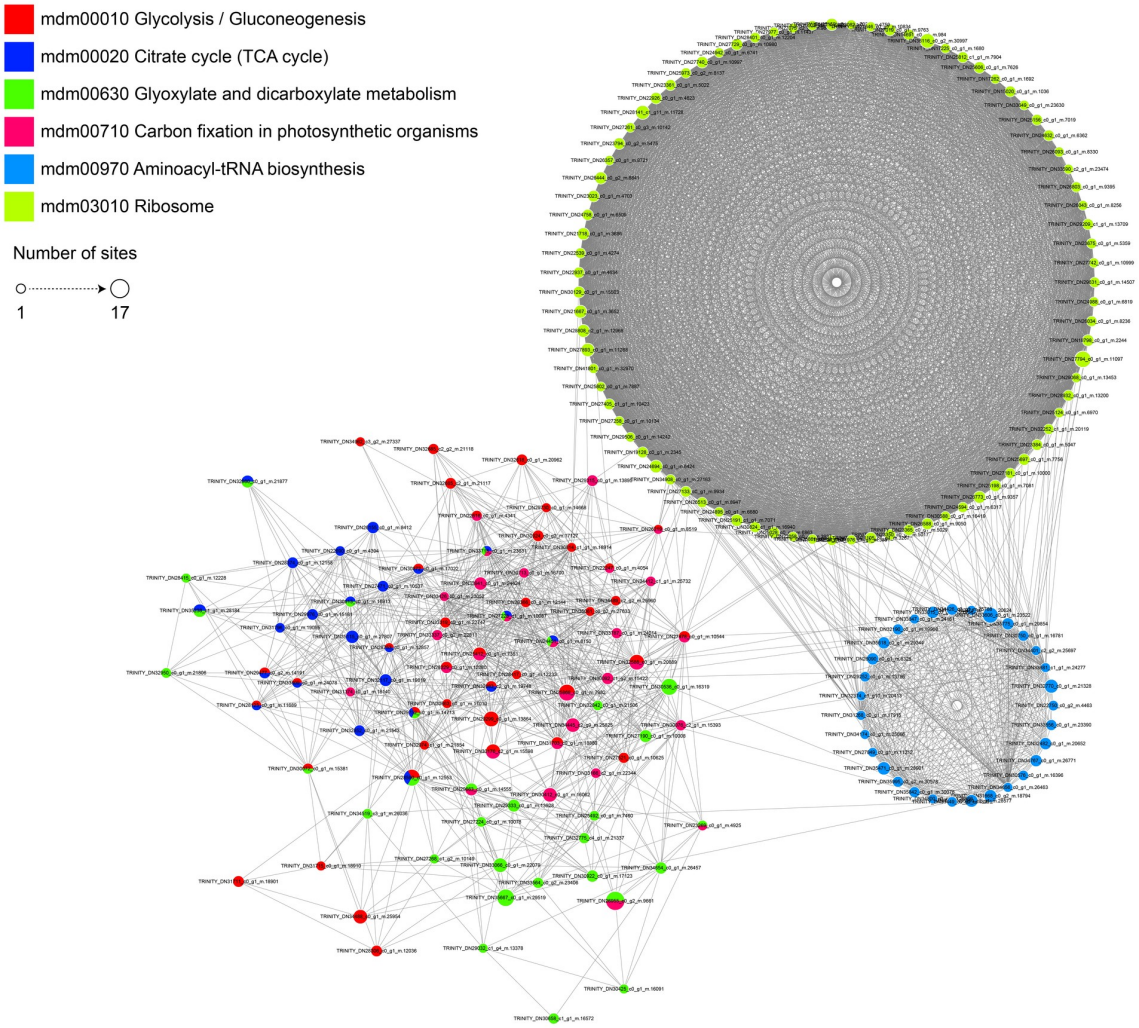
Protein-protein interaction network for proteins corresponding to modification sites.

To better understand the biological pathways and visualize virtually all cellular processes [55], we further built a PPI network for acetylated proteins (Fig 6, S4 Table). The detailed interaction of acetylation regulation processes and modified proteins can be deeply investigated in depth from this network (S4 Table**)**. As shown in the figure (Fig 6), a large and complex acetylated PPI network was constructed based on the number of proteins. In total, 1071 acetylated proteins were connected with one another; among them, 91 acetylated proteins were defined as nodes. The top cluster was the ribosome network, which consisted of 84 ribosome-associated proteins. Thirty-one amino-tRNA biosynthesis-related proteins were contained in the cluster II, while the remaining proteins were grouped as glycolysis/gluconeogenesis, the citrate cycle (TCA cycle), glyoxylate and dicarboxylate metabolism, and carbon fixation in photosynthetic organisms. Node degree is a key parameter for evaluating proteins in the network; among them, 4 proteins displayed the highest degree (≥40, S4 Table) and were located in chloroplasts, indicating that these proteins are dominant in chloroplasts. These results indicate that acetylated proteins are involved in a broad protein interaction network in paper mulberry.

### Acetylation associated with stress adaptation and protein in paper mulberry

Considering the challenging environment of the karst landform where the paper mulberry is located, such as drought and lack of nutrients, we specifically analyzed the acetylated proteins that may directly respond to stress. Based on the annotation, PAL (Phenylalanine ammonia-lyase), HSP70 (heat shock protein 70), and ERF (Ethylene-responsive transcription factor) were identified. The PAL family in the plants Arabidopsis (*Arabidopsis thaliama*), poplar (*Populus trichocarpa*) and rice (*Oryza sativa*) is composed of 4, 5 and 9 members, respectively, and they play an important role in resisting external environmental stress [56–58]. Moreover, HSP70 and ERF are both well-known for their function in cell response to stresses, such as those imposed by thermal stimuli and osmotic pressure [59–61].

**Fig 7:**
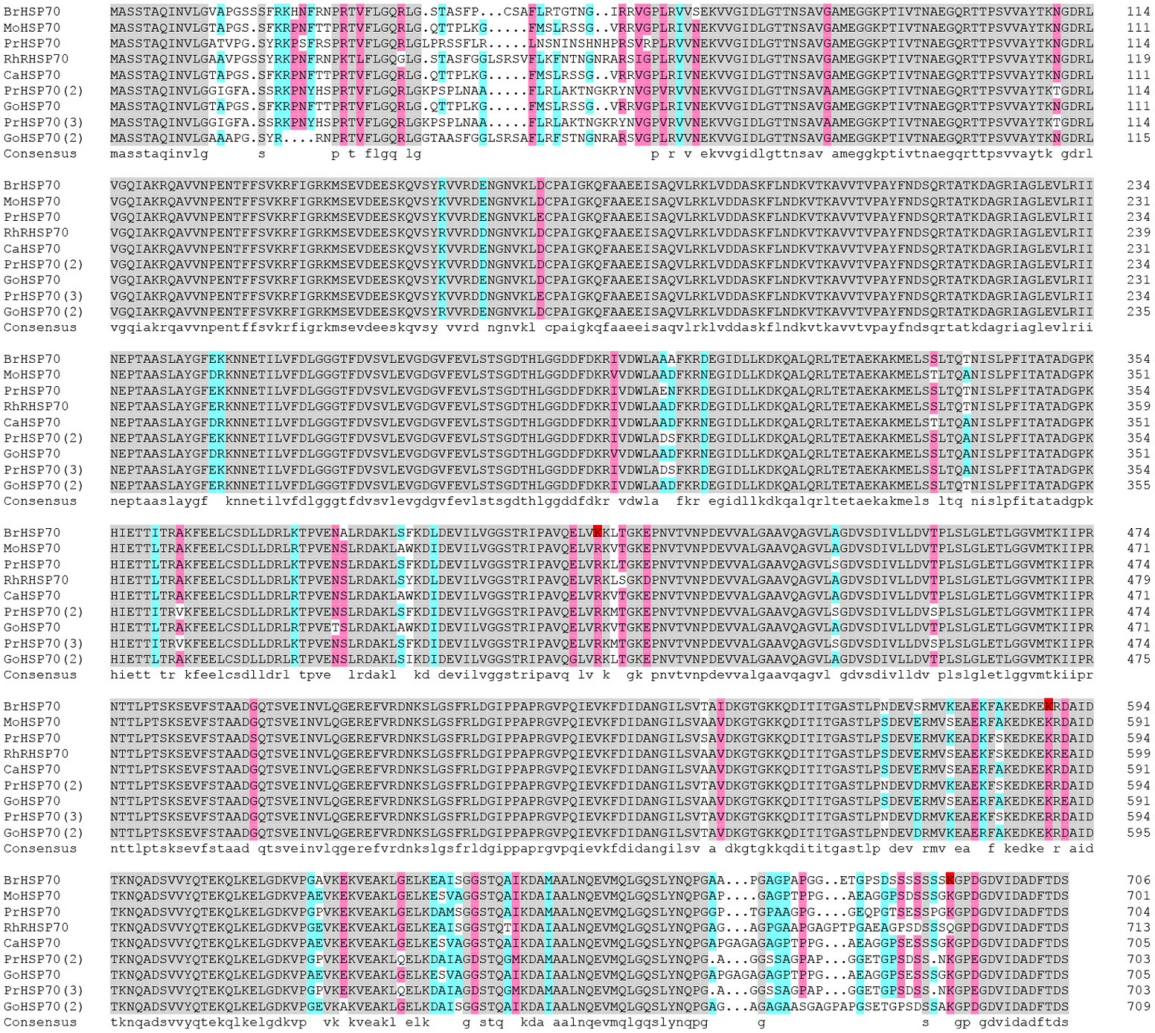
Sequence alignment of HSP70 amino acid among *Broussonetia papyrifera* and various selected species.

It has been discovered this year that HSP70 is mainly distributed in the cytoplasm, endoplasmic reticulum, mitochondria and chloroplast [62]. It is an important protein in plant abiotic and disease stress response. At the present, a large number of studies have shown that temperature is closely related to HSP70 expression. Drought stress can promote HSP70 expression in wheat, rice, maize and *Eupatorium adenophorum* [63–66], indicating that HSP70 may play a role as a molecular chaperone under adverse conditions to improve the ability of plants to cope with adverse environments by maintaining the stable conformation of proteins related to plant growth and development [67]. However, there is little evidence of HSP70 expression in woody plants. In this study, HSP70 acetylation sites were found, displayed 12 sites, and the deduced amino acids (aa) sequence contained 706 aa. Comparison on this protein’s sequences in other nine plants showed an overall consistency is 94.4% (Fig 7). The protein amino acid sequence conservation at the N-terminus is lower than that at the C-terminus, but there is a highly conserved motif structure (VIDADEFDS) at the C-terminus. In this study, the acetylated protein BrHSP70 is a very important member of the HSP family, it exists widely in nature and is highly conserved. Although different species have significant evolutionary differences, the evolution of HSP70 genes in plants is conserved [68]. Both proteins have an N-terminal accounting binding region and a C-terminal substrate binding region. There is an approximately 45kDa accounting binding region at the N terminal, that can hydrolyze APT and an approximately 25kDa substrate binding region at the C terminal can be exposed to the outside of the polypeptide substrate. The unfolded hydrophobic domain binds specifically, and SBD and NBD are connected by a twisted chain structure to function as a molecular chaperone [69–71]. The acetylated of BrHSP70, a typical motif characteristic at the C-terminus, directs the proteins to the cytoplasm. It is speculated that BrHSP70 can regulate the physiological functions of cells under adversity stress and participate in the correct assembly of proteins as a molecular chaperone to maintain specific protein conformations. We can infer that this lysine acetylation probably participates in the specific adaptation to the arid environment in paper mulberry, but the real role needs to be further verified.

The sequences are from *Morus notabilis*, *Prunus yedoensis var*. *nudiflora*, *Rhamnella rubrinervis*, *Cannabis sativa*, *Prunus avium*, *Gossypium tomentosum*, *prunus mume*, and *Gossypium raimondii*. The acetylation sites in HSP70 of *Broussonetia papyrifera* are labeled with red.

## Conclusion

This study provides lysine acetylation data for paper mulberry in the karst region. A total of 7,130 acetylation sites were identified on the 3179 proteins. The acetylation sites are located in different organelles and involved in various processes. Abundant lysine acetylation was discovered in the chloroplast, cytoplasm and nucleus. The acetylation of the key stress resistance protein HSP70 in the woody plant paper mulberry was clearly characterized for the first time. In addition, our method may be applicable to the comprehensive determination of lysine acetylation in other plants.

## Supporting information

S1 Table Information for the acetylated proteins in *Broussonetia papyrifera*. (XLSX)

S2 Table Modified site feature sequence and its enrichment statistics from MoMo software. (XLSX)

S3 Table Functional characterization and cellular localization of lysine acetylated proteins. (XLSX)

S4 Table Information for the protein-protein interaction network in *Broussonetia papyrifera*. (XLSX)

## References

[1] Zhen S, Deng X, Wang J, et al. First Comprehensive Proteome Analyses of Lysine Acetylation and Succinylation in Seedling Leaves of Brachypodium distachyon L. Scientific Reports, 2016, 6:31576. 10.1038/srep31576 27515067 PMC4981852

[2] Sheng-Bao S, Jian-Ding Q, Shao-Ping S, et al. Position-Specific Analysis and Prediction for Protein Lysine Acetylation Based on Multiple Features[J]. Plos One, 2012, 7(11): e49108. 10.1371/journal.pone.0049108 23173045 PMC3500252

[3] Huang He, Sabari Benjamin R., Garcia Benjamin A., Allis C. David, Zhao Yingming. SnapShot: Histone Modification. Cell,2014,159(2). 10.1016/j.cell.2014.09.037 PMC4324475 25303536

[4] He H, Shu L, Garcia BA, Yingming Z. Quantitative proteomic analysis of histone modifications. Chem Rev. 2015;115(6):2376–418. 10.1021/cr500491u 25688442 PMC4502928

[5] Sun J, Qiu C, Qian W. Ammonium triggered the response mechanism of lysine crotonylome in tea plants. BMC Genomics, 2019, 20(1). 10.1186/s12864-019-5716-z 31060518 PMC6501322

[6] Alicia Lundby, Kasper Lage, Brian, et al. Proteomic Analysis of Lysine Acetylation Sites in Rat Tissues Reveals Organ Specificity and Subcellular Patterns. Cell Reports,2012,2(2). 10.1016/j.celrep.2012.07.006 PMC4103158 22902405

[7] Hu L I, Lima B P, Wolfe A J. Bacterial protein acetylation: the dawning of a new age. Molecular Microbiology, 2010, 77(1):15–21. 10.1111/j.1365-2958.2010.07204.x 20487279 PMC2907427

[8] Jones J D, O’Connor C D. Protein acetylation in prokaryotes. Proteomics, 2011, 11(15):3012–3022. 10.1002/pmic.201000812 21674803

[9] Chen Z, J. Roles of dynamic and reversible histone acetylation in plant development and polyploidy. Biochimica Et Biophysica Acta, 2007. 10.1016/j.bbaexp.2007.04.007 17556080 PMC1950723

[10] Diaz-Vivancos P, Rubio M, Mesonero V, et al. The apoplastic antioxidant system in Prunus: response to long-term plum pox virus infection. Journal of Experimental Botany, 2006, 57(14):3813–3824. 10.1093/jxb/erl138 17043083

[11] Neilson K A, Mariani M, Haynes P A. Quantitative proteomic analysis of cold-responsive proteins in rice. Proteomics, 2011, 11(9):1696–1706. 10.1002/pmic.201000727 21433000

[12] Nallamilli BRR, Edelmann MJ, Zhong X, Tan F, Mujahid H, et al. Global Analysis of Lysine Acetylation Suggests the Involvement of Protein Acetylation in Diverse Biological Processes in Rice (Oryza sativa). PloS one. 2014,9(2):e89283. 10.1371/journal.pone.0089283 24586658 PMC3930695

[13] Barkla B J, Vera-Estrella R, Pantoja O. Progress and challenges for abiotic stress proteomics of crop plants. Proteomics, 2013, 13(12–13):1801–1815. 10.1002/pmic.201200401 23512887

[14] Weinert B T, Wagner S A, Horn H, et al. Proteome-Wide Mapping of the Drosophila Acetylome Demonstrates a High Degree of Conservation of Lysine Acetylation. Science Signaling, 2011, 4(183): ra48–ra48. 10.1126/scisignal.2001902 21791702

[15] Bharathi S S, Zhang Y, Mohsen A W, et al. Sirtuin 3 (SIRT3) Protein Regulates Long-chain Acyl-CoA Dehydrogenase by Deacetylating Conserved Lysines Near the Active Site. Journal of Biological Chemistry, 2013, 288(47):33837–33847. 10.1074/jbc.M113.510354 24121500 PMC3837126

[16] Still A J, Floyd B J, Hebert A S, et al. Quantification of Mitochondrial Acetylation Dynamics Highlights Prominent Sites of Metabolic Regulation. Journal of Biological Chemistry, 2013, 288(36):26209–26219. 10.1074/jbc.M113.483396 23864654 PMC3764825

[17] Xiong Y, Peng X, Cheng Z, et al. A comprehensive catalog of the lysine-acetylation targets in rice (Oryza sativa) based on proteomic analyses. Journal of Proteomics, 2016. 10.1016/j.jprot.2016.01.019 26836501

[18] Manosalva P M, Bruce M, Leach J E. Rice 14-3-3 protein (GF14e) negatively affects cell death and disease resistance. 2011, 68(5):777–787. 10.1111/j.1365-313X.2011.04728.x 21793954

[19] Denison FC, Paul AL, Zupanska AK, Ferl RJ. 14-3-3 proteins in plant physiology. Semin Cell Dev Biol. 2011;22(7):720–727. 10.1016/j.semcdb.2011.08.006 21907297

[20] Jiang J, Gai Z, Wang Y, et al. Comprehensive proteome analyses of lysine acetylation in tea leaves by sensing nitrogen nutrition. BMC Genomics, 2018, 19(1). 10.1186/s12864-018-5250-4 30477445 PMC6258439

[21] Xu YX, Chen W, Ma CL, et al. Proteome and Acetyl-Proteome Profiling of Camellia sinensis cv. ’Anjin Baicha’ during Periodic Albinism Reveals Alterations in Photosynthetic and Secondary Metabolite Biosynthetic Pathways. Front Plant Sci. 2017,8:2104. 10.3389/fpls.2017.02104 29312376 PMC5732353

[22] Xue B, Jeffers V, S W J Jr, et al. Protein intrinsic disorder in the acetylome of intracellular and extracellular Toxoplasma gondii. Molecular BioSystems, 2013, 9(4):645–657. 10.1039/c3mb25517d 23403842 PMC3594623

[23] Xiaolong Li, Xin Hu Yujing Wan, et al. Systematic Identification of the Lysine Succinylation in the Protozoan Parasiter Toxoplasma gondii. Journal of Proteome Research,2014, 13(12):6087–6095. 10.1021/pr500992r 25377623

[24] Pan J, Ye Z, Cheng Z, et al. Systematic Analysis of the Lysine Acetylome in Vibrio parahemolyticus. Journal of Proteome Research, 2014, 13(7):3294–3302. 10.1021/pr500133t 24874924

[25] Yoo H, Widhalm J R, Qian Y, et al. An alternative pathway contributes to phenylalanine biosynthesis in plants via a cytosolic tyrosine:phenylpyruvate aminotransferase. Nature Communications, 2013, 4. 10.1038/ncomms3833 24270997

[26] Xin Li, Longxiang, et al. Unexpected extensive lysine acetylation in the trump-card antibiotic producer Strcptomyces roseosporus revealed by proteome-wide profiling. Journal of Proteomics, 2014. 10.1016/j.jprot.2014.04.017 24768905

[27] Jiang J, Gai Z, Wang Y, et al. Comprehensive proteome analyses of lysine acetylation in tea leaves by sensing nitrogen nutrition[J]. BMC Genomics, 2018, 19(1). 10.1186/s12864-018-5250-4 30477445 PMC6258439

[28] Choudhary C, Kumar C, Gnad F, et al. Lysine acetylation targets protein complexes and co-regulates major cellular functions. Science. 2009;325(5942):834–840. 10.1126/science.1175371 19608861

[29] xiangming XU, hechun YU, Guofeng LI. progress in research of plant tolerance to saline stress. Chinese journal of applied and environment biology,2000,6(4):379–387.

[30] Kaiser W M. Effects of water deficit on photosynthetic capacity[J]. Physiologia Plantarum, 2010, 71(1):142–149.

[31] Cornic G, Briantais J M. Partitioning of photosynthetic electron flow between CO2 and O 2 reduction in a C 3 leaf (Phaseolus vulgaris L.) at different CO 2 concentrations and during drought stress.[J]. Planta, 1991, 183(2):178–184. 10.1007/BF00197786 24193618

[32] Buckland S M, Hendry P G A F. The Role of Ascorbate in Drought-Treated Cochlearia atlantica Pobed. and Armeria maritima (Mill.) Willd[J]. New Phytologist, 1991, 119(1):155–160. 10.1111/j.1469-8137.1991.tb01019.x 33874334

[33] Price A H, Hendry G A F. Iron-catalysed oxygen radical formation and its possible contribution to drought damage in nine native grasses and three cereals[J]. Plant Cell & Environment, 2010, 14(5):477–484.

[34] Seel W E, F H G A, Lee J A. Effects of desiccation on some activated oxygen processing enzymes and anti-oxidants in mosses[J]. Journal of Experimental Botany(8):1031–1037.

[35] Zhang J, Sprung R, Pei J, et al. Lysine acetylation is a highly abundant and evolutionarily conserved modification in Escherichia coli. Mol Cell Proteomics. 2009;8(2):215–225. 10.1074/mcp.M800187-MCP200 18723842 PMC2634580

[36] Choudhary C, Kumar C, Gnad F, et al. Lysine acetylation targets protein complexes and co-regulates major cellular functions. Science. 2009;325(5942):834–840. 10.1126/science.1175371 19608861

[37] Asai Akieda, Zaima Sayaka, Ikegami Nobuhiro, Kahyo Koji, Yao Tomoaki, Hatanaka Ikuko, et al. SIRT1 Regulates Thyroid-Stimulating Hormone Release by Enhancing PIP5Kγ Activity through Deacetylation of Specific Lysine Residues in Mammals. PloS one. 2010. 10.1371/journal.pone.0011755 PMC2909264 20668706

[38] Weinert B T, Wagner S A, Horn H, et al. Proteome-Wide Mapping of the Drosophila Acetylome Demonstrates a High Degree of Conservation of Lysine Acetylation[J]. Science Signaling, 2011, 4(183):48. 10.1126/scisignal.2001902 21791702

[39] Chunaram Choudhary, Chanchal Kumar, Florian Gnad, et al. Lysine acetylation targets protein complexes and co-regulates major cellular functions.[J]. Science (New York, N.Y.),2009,325(5942):834–40. 10.1126/science.1175371 19608861

[40] Mooney H, A. The Carbon Balance of Plants[J]. Annual Review of Ecology and Systematics, 1972.

[41] Walters M. B., Kruger E. L., Reich P. B.. Relative Growth Rate in Relation to Physiological and Morphological Traits for Northern Hardwood Tree Seedlings: Species, Light Environment and Ontogenetic Considerations. 1993, 96(2):219–231. 10.1007/BF00317735 28313418

[42] Mcallister C A, Maragni K L A. Is Leaf-Level Photosynthesis Related to Plant Success in a Highly Productive Grassland. Oecologia, 1998, 117(1–2):40–46. 10.1007/s004420050629 28308504

[43] Knee M, Thomas L C. Light utilization and competition between Echinacea purpurea, Panicum virgatum and Ratibida pinnata under greenhouse and field conditions. Ecological Research, 2002, 17.

[44] Baker N R. A possible role for photosystem II in environmental perturbations of photosynthesis. Physiologia Plantarum, 1991, 81(4):563–570.

[45] Finkemeier Iris, Laxa Miriam, Miguet Laurent, et al. Proteins of Diverse Function and Subcellular Location Are Lysine Acetylated in Arabidopsis. 2011, 155(4):1779–1790. 10.1104/pp.110.171595 PMC3091095 21311031

[46] Nagel J M, Griffin K L. Can Gas-Exchange Characteristics help Explain the Invasive Success ofLythrum salicaria. Biological Invasions, 2004, 6(1):101–111.

[47] Zhang C, Liu W, Xu Z, et al. Responses of vegetative growth and photosynthesis to temperature in the invasive species Alternanthera philoxeroides and its indigenous congener A. sessilis. Journal of Tropical and Subtropical Botany, 2006.

[48] Min H, Jiao C, Zhongli S. First comprehensive analysis of lysine acetylation in Alvinocaris longirostris from the deep-sea hydrothermal vents[J]. Bmc Genomics, 2018, 19(1):352. 10.1186/s12864-018-4745-3 29747590 PMC5946511

[49] Song L, Wang G, Malhotra A, et al. Reversible acetylation on Lys501 regulates the activity of RNase II[J]. Nucleic Acids Research, 2016. 10.1093/nar/gkw053 26847092 PMC4797298

[50] Xiangyun Y, Xiaomin N, Linping G, et al. Desuccinylation of pyruvate kinase M2 by SIRT5 contributes to antioxidant response and tumor growth[J]. Oncotarget, 2016, 8(4):6984–6993. 10.18632/oncotarget.14346 PMC5351684 28036303

[51] Di Zhao, Shao-Wu Zou, Ying Liu, Xin Zhou, et al. Guan. Lysine-5 acetylation negatively regulates lactate dehydrogenase A and is decreased in pancreatic cancer.[J]. Cancer cell, 2013, 23(4):464–76. 10.1016/j.ccr.2013.02.005 23523103 PMC3885615

[52] Rardin Matthew J, Newman John C, Held Jason M, Cusack Michael P, Sorensen Dylan J, et al. Label-free quantitative proteomics of the lysine acetylome in mitochondria identifies substrates of SIRT3 in metabolic pathways.[J]. Proceedings of the National Academy of Sciences of the United States of America,2013,110(16):6601–6. 10.1073/pnas.1302961110 23576753 PMC3631688

[53] Gibson B W. The human mitochondrial proteome: oxidative stress, protein modifications and oxidative phosphorylation[J]. International Journal of Biochemistry & Cell Biology, 2005, 37(5):927–934. 10.1016/j.biocel.2004.11.013 15743667

[54] Qiulin Q. Molecylar Cloning and characterization of transcription factors involved in Lignin Biosynthetic pathway and phenylprolanoid pathway. Fudan University, 2007.

[55] Wang H, Huang H, Ding C, et al. Predicting Protein-Protein Interactions from Multimodal Biological Data Sources via Nonnegative Matrix Tri-Factorization.International Conference on Research in Computational Molecular Biology. Springer, Berlin, Heidelberg, 2012. 10.1089/cmb.2012.0273 23509857

[56] Tsai Chung-Jui, Harding S A, Tschaplinski T J, et al. Genome-wide analysis of the structural genes regulating defense phenylpropanoid metabolism in Populus.[J]. New Phytologist, 2010, 172. 10.1111/j.1469-8137.2006.01798.x 16945088

[57] Hamberger B, Ellis M, Friedmann M, et al. Genome-wide analyses of phenylpropanoid-related genes in Populus trichocarpa, Arabidopsis thaliana, and Oryza sativa: the Populus lignin toolbox and conservation and diversification of angiosperm gene families[J]. Canadian Journal of Botany, 2007, 85(12):1182–1201.

[58] Wong J H, Namasivayam P, Abdullah M P. The PAL2 promoter activities in relation to structural development and adaptation in Arabidopsis thaliana[J]. Planta, 2012, 235(2):267–277. 10.1007/s00425-011-1506-9 21874349

[59] Min H, Yuan L, Chengwen S, et al. Transcriptome Changes in Eriocheir sinensis Megalopae after Desalination Provide Insights into Osmoregulation and Stress Adaption in Larvae[J]. Plos One, 2014, 9(12):e114187. 10.1371/journal.pone.0114187 25470496 PMC4254945

[60] Zhang Y, Sun J, Mu H, et al. Proteomic Basis of Stress Responses in the Gills of the Pacific Oyster Crassostrea gigas[J]. Journal of Proteome Research, 2014, 14(1):304–317. 10.1021/pr500940s 25389644

[61] Kurzik-Dumke U, Lohmann E. Sequence of the new Drosophila melanogaster small heat-shock-related gene, lethal(2) essential for life [l(2) efl], at locus 59F4,5—ScienceDirect[J]. Gene, 1995, 154(2):171–175. 10.1016/0378-1119(94)00827-f 7890160

[62] Yan QI, Zhao-shi XU, Pan-song LI, Ming CHEN, Lian-cheng LI, You-zhi MA. Research Progress on Molecular Mechanism and Application of HSP70 in Plants. Journal of Plant Genetic Resources, 2013,14(3):507–511.

[63] Yan-ping XIAO, Wei-Na GONG, Fang-hao WAN, et al. Cloning and Sequence Analysis of Heat Shock Protein 70 Gene from Ageratina adenophora(Eupatorium adenophorum)[J], Journal of Agricultural Science Technology. 2010, 12(1):111–111.

[64] Yanqiu AN, Ruiming Liu, Jing Feng, et al. Molecular Cloning of Wheat Heat Shock Protein Gene HSP70 and Expression Analysis in Plant Defense and Stress Responses [J]. Molecule Plant Breeding, 2011, 009(004):402–409.

[65] Shang-zhi HUANG, Xiang-Fu HUANG, Xiao-Dong LIN, et al. Induction of Chilling Tolerance and Heat Shock Protein Synthesis in Rice Seedlings by Heat Shock [J]. Acta Photophysiologica Sinica,2004,30(2):189–194. 15599046

[66] Hui-cong LI, Xiu-lin GUO, Dong-mei WANG, Guo-liang LI. Responses of HSP70 gene expression to temperature stresses in maize (Zea mays L.). Journal of Agricultural University of Hebei, 2010,33(6):12–15.

[67] Baoyun FENG, Rong Li, Zhongxiong LAI, et al. Cloning and Expression Analysis of HSP70 in Oncidium hybridum [J]. Chinese Journal of Tropical Crops, 2020, v.41(04):119–128.

[68] Xu Chen, Lei Shi, Lu Zhu, et al. Molecular Evolution Characteristics and Expression Pattern Analysis of the Heat Shock Protein 70(HSP70) Gene Superfamily in Plant [J]. Cenomics and Applied Biology, 2017(10):370–382.

[69] Wilbanks S M, Delucaflaherty C, Mckay D B. Structural basis of the 70-kilodalton heat shock cognate protein ATP hydrolytic activity. I. Kinetic analyses of active site mutants.[J]. Journal of Biological Chemistry, 1994, 269(17):12893. 8175706

[70] Nelson John R, et al. The translation machinery and 70 kd heat shock protein cooperate in protein synthesis[J]. Cell, 1992, 71(1):97–105. 10.1016/0092-8674(92)90269-i 1394434

[71] Kang P J. Requirement for hsp 70 in the mitochondrial matrix for translocation and folding of precur-sor proterins.[J]. Nature, 1990, 348(6297):137–143. 10.1038/348137a0 2234077

